# A Fast Denoising Dataset-Generation Method for Ground Penetrating Radar Based on a Transformer Network Constrained by an Underground Clutter Physical Model

**DOI:** 10.3390/s26206501

**Published:** 2026-10-14

**Authors:** Yuxuan Wu, Xiaole Ma, Feng Shen, Yijie Wang

**Affiliations:** 1 The School of Instrumentation Science and Engineering, Harbin Institute of Technology, Harbin 150001, China; wuyuxuan@hit.edu.cn; 2 Suzhou Research Institute, Harbin Institute of Technology, Suzhou 215128, China; 3 Suzhou Foreign Language School, Suzhou 215129, China; sflsmaxiaole@126.com; 4 The School of Electrical Engineering and Automation, Harbin Institute of Technology, Harbin 150001, China; wangyijie@hit.edu.cn

**Keywords:** ground-penetrating radar, B-scan generation, denoising dataset, transformer network, underground clutter, physical model, data augmentation

## Abstract

Ground-penetrating radar (GPR) denoising requires large paired datasets that preserve weak target echoes while representing realistic underground clutter, but field measurements are difficult to label, and high-fidelity electromagnetic simulations are costly to scale. This paper proposes a fast denoising dataset-generation method based on a transformer network constrained by an underground clutter physical model. The method decomposes each generated B-scan into a noisy image, a clean target response, a target mask, and three physically interpretable clutter components: direct antenna coupling, random background clutter, and interface-induced multipath. A reference B-scan is used only to estimate gray-level, vertical-energy, and spectral statistics, while transformer modulation branches adjust the physically generated clutter components without copying reference image details. In a 100-sample experiment, the proposed generator produced 512×512 B-scans on a CUDA-enabled GPU in 38.36 s, corresponding to 2.61 B-scans/s and 15.64 exported maps/s. The dataset contained 600 maps, including 500 supervision or component maps. The mean reference correlation was −0.0210, indicating low pixel-level dependence on the reference image. Compared with representative GAN, diffusion, and forward-modeling generators, the proposed method provides explicit target and clutter labels for denoising-oriented dataset construction. Five matched gprMax/FDTD conditions yielded a direct-referenced propagation-delay MAPE of 4.70% and a hyperbola-curvature MAPE of 14.41%, while larger amplitude and multipath errors revealed the expected fidelity–efficiency trade-off of the simplified generator.

## 1. Introduction

Ground-penetrating radar (GPR) is a non-destructive detection technique for imaging shallow subsurface structures through electromagnetic wave reflection. Since it can provide B-scan profiles without excavation, it has been widely used in road and railway inspection, underground utility surveying, pipeline detection, subgrade distress identification, and robotic subsurface mapping. Recently, many studies show that intelligent GPR processing can support subsurface map generation from multi-array measurements [1], foundation-model-based distress segmentation [2], target-signature segmentation [3], and shape-aware hyperbola detection for buried pipelines [4]. However, they also reveal a central limitation: robust deep models require large, diverse, and accurately labeled data, whereas field GPR data are difficult to acquire, expensive to annotate, and strongly affected by site-dependent clutter, attenuation, antenna coupling, and target-structure variability.

This data-reconstruction problem is also shared by intelligent sensing systems outside GPR. In a digital-twin framework for structural condition assessment, Zhang et al. reconstructed heterogeneous displacement, strain, and acceleration responses from a subset of measured responses and a transmissibility model, demonstrating how physically coupled responses can be recovered when some sensing channels are unavailable [5]. A related non-contact vision-based study reconstructed structural responses from visual observations and used the recovered responses for substructural assessment [6]. These studies address measurement-side response reconstruction rather than forward generation of GPR B-scans, but they provide a useful broader perspective: a reconstructed response should preserve the physical relationships that are relevant to the downstream task and remain reliable under noisy or incomplete observations. For GPR dataset construction, the corresponding requirement is to preserve target arrival geometry and interpretable clutter components while varying acquisition and subsurface conditions. This motivates the combination of physical constraints and data-driven modulation adopted in this work, and it also highlights the need for cross-condition validation rather than relying only on image-level similarity.

One direct way to enlarge GPR datasets is electromagnetic forward simulation. Numerical methods, such as finite-difference time-domain (FDTD) and Finite Element Method (FEM), can generate labeled examples under controlled dielectric properties, target geometries and acquisition settings. In addition, in 2022, a statistical finite-difference frequency-domain (FDFD) simulation method has been used to build training data for tasks such as humanitarian demining [7]. Simulation-based data are physically interpretable and can provide labels that are unavailable in field measurements. However, high-fidelity electromagnetic modeling is computationally expensive when the scene contains heterogeneous media, rough interfaces, dense parameter sweeps, or many target types. This cost becomes more restrictive when clean and noisy paired samples are needed for denoising or when the dataset must cover a broad distribution of underground clutter. Therefore, data generation methods for GPR must balance physical credibility, computational efficiency, and diversity.

Generative adversarial networks (GANs) have been widely explored to reduce the dependence on costly measurements and simulations. WAEGAN introduced a GAN-based augmentation strategy for GPR data [8], while GPR-GAN investigated a dedicated generative adversarial framework for GPR data generation in pavement and subgrade scenarios [9]. Multiscale discrimination has also been adopted to improve B-scan augmentation quality by constraining features at different spatial resolutions [10]. More recent studies have introduced feature-fusion and conditional designs, such as CIGGAN for GPR image generation [11] and improved DC-GAN models for target-oriented image synthesis [12]. In addition, FM-GAN embeds forward-modeling information and transformer tuning to generate pipeline B-scans with improved realism [13]. These GAN-based methods are efficient at inference and useful for augmentation, but adversarial training can suffer from instability, mode collapse, and weak controllability over the physical sources of clutter. As a result, generated B-scans may be visually plausible while still failing to preserve the signal mechanisms required by downstream denoising or interpretation models.

Diffusion models have recently become an important alternative because they can learn complex data distributions through iterative denoising. DDPM-based GPR image generation has been investigated as a comprehensive synthetic-data pipeline [14], and VMD-guided diffusion has been used to exploit modal features of GPR signals for road underground targets [15]. Forward-guided and reverse-resampling diffusion further combines restoration and generation for GPR B-scan data [16]. Compared with GANs, diffusion models generally provide stable training and strong sample quality. Nevertheless, the repeated sampling steps increase generation time, and the learned denoising process is often driven primarily by data statistics rather than explicit underground clutter physics. For large-scale denoising dataset construction, this trade-off limits practical efficiency, especially when many noise levels, target depths, and soil conditions must be represented.

To sum up, there are still three unresolved issues in existing research. Firstly, purely simulation-based datasets can be physically meaningful but slow to scale. Secondly, GAN-based augmentation is fast but may not provide sufficient physical control or training stability. Thirdly, diffusion-based generation improves fidelity but introduces high sampling cost. For GPR denoising, these issues are coupled with a further requirement: the generated data should preserve target echo structures while representing realistic clutter interference, so that the denoising model learns to suppress background noise without removing weak subsurface targets. This requirement is not fully addressed by general image-generation pipelines or by target-detection-oriented augmentation methods.

To solve this problem, we propose a fast denoising dataset-generation method for GPR based on a transformer network constrained by an underground clutter physical model. The motivation is to combine the long-range feature modeling capability of transformers with physical prior constraints, which describe clutter behavior in underground media. On this basis, the generation process is expected to improve efficiency while maintaining physically meaningful target-clutter relationships. The resulting framework is intended to support the construction of denoising-oriented GPR datasets and provide reliable training samples for subsequent intelligent interpretation tasks.

In this paper, noise denotes random, usually incoherent amplitude variation introduced by receiver and mixer electronics, transmitter timing or amplitude jitter, and wideband interference entering the antenna. Noise is not tied to a reproducible subsurface geometry and is evaluated through the signal-to-noise ratio (SNR). Clutter denotes unwanted but spatially or temporally structured responses that can resemble a target, including geological-interface reflections, antenna-internal reflections, direct antenna coupling, and delayed or virtual target paths caused by multipath interactions. Clutter is evaluated through the signal-to-clutter ratio (SCR) and requires geometry-aware, model-based, or adaptive separation. Thus, the terms are kept distinct below: $N_{i}$ denotes stochastic noise, whereas $C_{i}$ denotes structured clutter. A generated field containing both is called a composite background field.

## 2. Underground Clutter Physical Model

**Figure 1:**
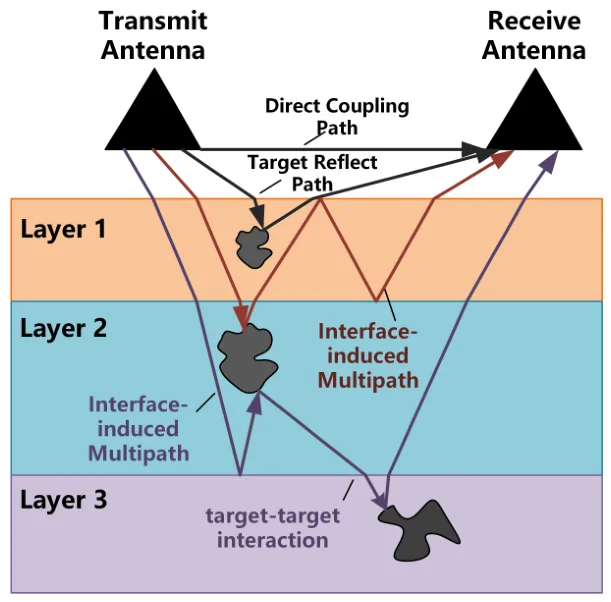
Multipath signal distribution in multilayer soil.

In a multilayer underground medium, the characteristics of GPR echoes are not only determined by the target response but also by several non-target electromagnetic paths. As is shown in Figure 1, according to the multilayer clutter model, the interfaces between adjacent soil layers are assumed to be locally smooth. Thus, the incident wave undergoes specular reflection and refraction at each interface. On that basis, the collected B-scan can be decomposed into four physically meaningful components: direct antenna coupling, direct target reflection, interface-induced multipath, and random target-target interaction. Direct antenna coupling is produced by the signal traveling directly from the transmitting antenna to the receiving antenna. Direct target reflection denotes the wave path that reaches the underground target and returns to the receiver. Interface-induced multipath is generated by repeated reflections at soil-layer boundaries and can occur on both the transmitting and receiving sides. When multiple buried objects exist, random mutual reflections between targets may produce false target-like responses.

For direct antenna coupling, the signal propagates approximately along a straight path in air. Therefore, its delay is determined by the antenna spacing

(1) $$\tau_{\mathrm{dc}}=\frac{d_{a}}{c},$$

where $d_{a}$ is the distance between the transmitting and receiving antennas, and *c* is the speed of light in free space. The corresponding free-space attenuation can be written as

(2) $$A_{\mathrm{dc}}=20\log_{10}\left(\frac{1}{d_{a}}\right),$$

which describes the spherical spreading loss of the directly coupled field. Since this component appears at early time gates and is highly coherent along the scan direction, it forms a strong horizontal clutter band in B-scan images.

For the direct target-reflection path, the electromagnetic wave crosses several media before reaching the buried object. At the interface between the *n*-th and (n+1)-th layers, Snell’s law gives

(3) $$\frac{\sin\theta_{n}}{\sin\theta_{n+1}}=\sqrt{\frac{\varepsilon_{r,n+1}}{\varepsilon_{r,n}}}=\frac{\eta_{n+1}}{\eta_{n}},$$

where $\theta_{n}$ is the propagation angle in the *n*-th layer, $\varepsilon_{r,n}$ is the relative permittivity, and $\eta_{n}$ is the refractive index. If the target is located in the *N*-th layer at depth *d*, the one-way propagation delay from the antenna side to the target can be modeled as

(4) $$\tau_{\mathrm{tar}}=\frac{h_{0}}{c\cos\theta_{0}}+\sum_{n=1}^{N-1}\frac{h_{n}\sqrt{\varepsilon_{r,n}}}{c\cos\theta_{n}}+\frac{\left(d-\sum_{n=1}^{N-1}h_{n}\right)\sqrt{\varepsilon_{r,N}}}{c\cos\theta_{N}},$$

where $h_{0}$ is the antenna height above the ground surface, and $h_{n}$ is the thickness of the *n*-th soil layer. In the B-scan domain, lateral antenna motion changes the geometrical distance to the target, so this direct reflection produces a hyperbolic target signature.

Interface-induced multipath is modeled by adding extra reflected path segments to the direct target path. For receiving-side multipath, the target echo may enter an upper layer and then be repeatedly reflected by a soil interface before it reaches the receiving antenna. If the echo is reflected $n_{r}$ times inside the *i*-th layer, a compact delay model can be written as

(5) $$\tau_{\mathrm{rx}}^{\left(n_{r}\right)}=\tau_{\mathrm{tar}}+\frac{2n_{r}h_{i}\sqrt{\varepsilon_{r,i}}}{c\cos\theta_{i}}.$$

**Figure 2:**
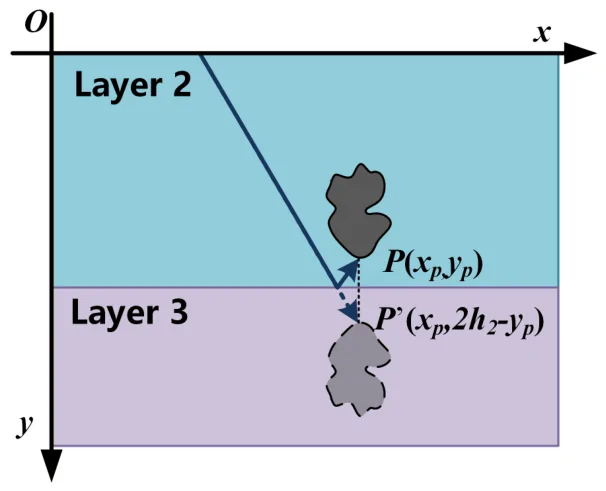
Multipath geometric model on the transmitting side.

This term explains why multipath harmonics often appear as a set of delayed and approximately equally spaced responses in the range direction. As is shown in Figure 2, for transmitting-side multipath, an interface reflection before target illumination can be represented by an image target. If the real target is located at $P=(x_{p},y_{p})$ and the reflecting boundary is associated with a layer thickness $h_{2}$, the virtual target is

(6) $$P^{\prime }=\left(x_{p},2h_{2}-y_{p}\right).$$

The reflected transmitting path is then evaluated by replacing the real target position with $P^{\prime }$ in the propagation-distance calculation. These virtual paths generate false or shifted target-like structures and are a major source of structured underground clutter.

The energy loss at soil interfaces is governed by Fresnel reflection. For a dielectric-dielectric boundary, the reflected energy ratio is

(7) $$F\left(\theta_{n}\right)=\frac{1}{2}\left(R_{s}+R_{p}\right),$$

where the perpendicular and parallel polarization reflection coefficients are

(8) $$\begin{array}{cc} R_{s} & ={\left|\frac{\eta_{n}\cos\theta_{n}-\eta_{n+1}\cos\theta_{n+1}}{\eta_{n}\cos\theta_{n}+\eta_{n+1}\cos\theta_{n+1}}\right|}^{2}, \end{array}$$

(9) $$\begin{array}{cc} R_{p} & ={\left|\frac{\eta_{n}\cos\theta_{n+1}-\eta_{n+1}\cos\theta_{n}}{\eta_{n}\cos\theta_{n+1}+\eta_{n+1}\cos\theta_{n}}\right|}^{2}. \end{array}$$

The refracted angle is constrained by

(10) $$\cos\theta_{n+1}=\sqrt{1-{\left(\frac{\eta_{n}}{\eta_{n+1}}\sin\theta_{n}\right)}^{2}}.$$

When the term under the square root becomes negative, total reflection occurs, and the reflected energy ratio is taken as F=1.

For a metal-medium boundary, the complex refractive behavior of the target can be described by introducing the extinction coefficient $k_{t}$ and the complex refractive index $\eta_{t}-jk_{t}$. In the GPR frequency band, metallic targets usually exhibit very high reflectivity, and the target reflection coefficient can therefore be approximated as

(11) $$F_{t}\approx 1.$$

For non-metallic targets, the target reflectivity is calculated in the same way as a dielectric interface.

Based on the above reflection and transmission terms, the attenuation of a direct target-reflection path can be expressed in decibels as

(12) $$A_{\mathrm{tar}}=20\log_{10}\left(\frac{1}{r}\right)+\sum_{i\in T_{\mathrm{tx}}}10\log_{10}\left(1-F_{i}\right)+\sum_{j\in T_{\mathrm{rx}}}10\log_{10}\left(1-F_{j}\right)+10\log_{10}F_{t},$$

where *r* is the total propagation length, $T_{\mathrm{tx}}$ and $T_{\mathrm{rx}}$ denote the interface transmission events on the transmitting and receiving sides, and $F_{t}$ is the target reflection coefficient. For multipath propagation, additional interface reflection losses must be included

(13) $$A_{\mathrm{mp}}=A_{\mathrm{tar}}+\sum_{q=1}^{m}10\log_{10}F_{n(q)},$$

where *m* is the number of multipath reflections, and $F_{n(q)}$ is the reflection coefficient of the interface where the *q*-th multipath reflection occurs. This formulation provides the physical constraint used in this work: generated B-scan data should preserve the target hyperbola while explicitly containing early-time direct coupling, stochastic background noise, and delayed interface-induced multipath responses.

## 3. Fast Denoising Dataset-Generation Method

The target classes considered in the current dataset are compact, high-contrast subsurface objects that generate hyperbola-like diffraction signatures in a B-scan, including metallic pipes, cables, rebars, and compact dielectric inclusions or void boundaries. In the generator, these targets are represented by lateral position, normalized depth, hyperbola curvature, echo width, polarity, amplitude, and an effective reflection strength. Therefore, the proposed synthesis focuses on a controlled target-response model for compact diffraction signatures rather than a complete taxonomy of all possible underground objects.

The nominal acquisition profile contains an air gap above a near-surface wall or soil layer and a deeper cavity/target region. The parameters listed below define the reproducible simulation profile used by the generator. The relative permittivity is treated as an effective medium parameter for controlled synthesis; therefore, site-specific effects such as moisture-dependent permittivity, conductivity variation, and attenuation should be calibrated when the method is applied to a particular survey area.

Dataset scale and validation scope. The quantitative results reported in this manuscript are based on one 100-sample synthetic run. The current run uses the nominal acquisition geometry and effective medium parameters listed in Table 1; target position, depth, response shape, clutter realization, and component gains are varied within this configuration. The five frequency-band examples reported below are a qualitative frequency sweep and should not be interpreted as an independent cross-frequency generalization experiment. Finally, we additionally reports model-based estimates at the moisture contents of three selected test areas, but the 100-sample run does not constitute an independent validation across those areas, soil profiles, or antenna systems. We therefore restrict the current claims to the reproducibility and component-level validity of the 100-sample generation run, and do not claim that these results establish site-independent generalization.

The proposed dataset generator is implemented as a physics-constrained transformer framework for constructing paired noisy and target-preserving GPR B-scan samples. The method is designed for denoising data preparation rather than direct target detection. Each generated sample contains a noisy B-scan image, a clean target-response map, a target mask, and three explicitly separated clutter components: direct antenna coupling, random background clutter, and interface-induced multipath. The generator is implemented in PyTorch and supports batched CPU or GPU execution. In the current implementation, a reference B-scan image is not copied as a pixel-level template. Instead, it is used only to estimate global grayscale range, vertical energy distribution, and spectral characteristics, so that the generated data remain visually realistic without becoming a duplicate of a single measured profile.

**Figure 3:**
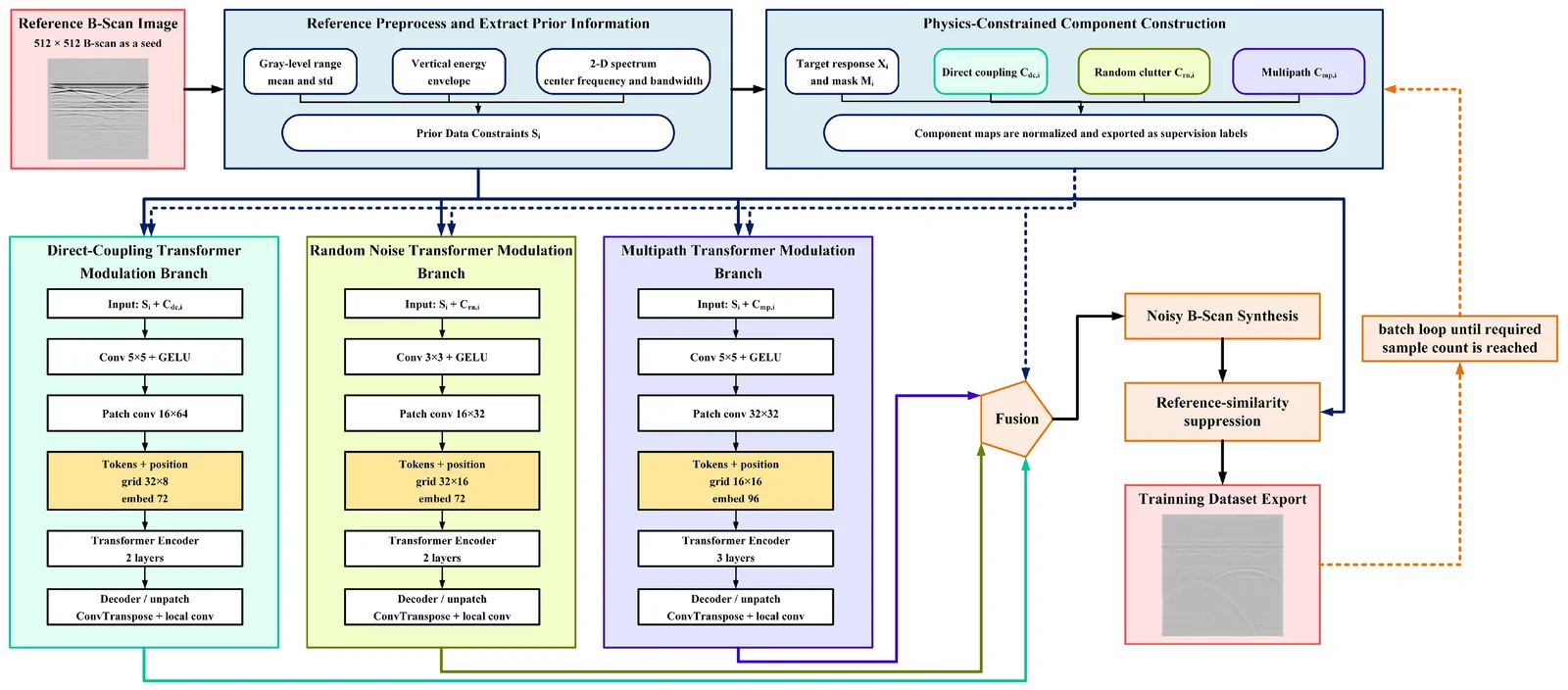
Workflow of the proposed fast denoising dataset-generation method. The generator uses a reference B-scan only to extract statistical style priors, constructs target and clutter components under the underground clutter model, modulates the physically generated direct-coupling, stochastic-background, and multipath fields through three transformer branches, and finally exports a structured denoising dataset with noisy images, clean target responses, target masks, separated clutter maps, modulation maps, and diagnostic metadata.

Figure 3 illustrates the complete data construction pipeline. The left part represents the input reference B-scan and run configuration, which provide grayscale, vertical-energy, and spectral constraints but are not used as pixel-level templates. The middle part shows the generation of physically interpretable target, direct-coupling, stochastic-background, and multipath components, followed by branch-specific transformer modulation with patch sizes matched to the spatial characteristics of each clutter source. The right part summarizes fusion, noisy B-scan synthesis, reference-similarity suppression, and dataset export. This design links the physical clutter model to the neural modulation process and explains why each generated sample contains not only a noisy B-scan but also supervision maps that can be directly used for target-preserving denoising.

The overall generation pipeline is introduced as follows. Let $I_{\mathrm{ref}}\in {\left[0,1\right]}^{H\times W}$ denote the reference grayscale B-scan. For each mini-batch, the generator first extracts reference-statistical priors from $I_{\mathrm{ref}}$ and then samples physically interpretable target and clutter fields. These fields are processed by three transformer modulation branches and a lightweight fusion head to produce the final noisy B-scan. The generated sample can be represented as

(14) $$D_{i}=\left\{Y_{i},\;X_{i},\;M_{i},\;C_{i}^{\mathrm{dc}},\;C_{i}^{\mathrm{rn}},\;C_{i}^{\mathrm{mp}}\right\},$$

where $Y_{i}$ is the generated noisy B-scan, $X_{i}$ is the normalized clean target response, $M_{i}$ is the target mask, and $C_{i}^{\mathrm{dc}}$, $C_{i}^{\mathrm{rn}}$, and $C_{i}^{\mathrm{mp}}$ denote direct coupling, a stochastic background noise/interference field, and structured multipath clutter, respectively. This explicit decomposition makes the generated samples suitable for supervised denoising, component analysis, and ablation experiments.

In the generator, to preserve realistic signal appearance while avoiding overfitting to a single reference image, the method extracts only low-dimensional statistics from $I_{\mathrm{ref}}$. The reference mean and standard deviation define the gray-level range. A vertical energy envelope is obtained by averaging the reference image along the scan direction and smoothing it along the range direction. In addition, the center frequency and bandwidth of the high-frequency reference residual are estimated from the two-dimensional power spectrum

(15) $$P\left(f_{x},f_{y}\right)={\left|F\left(I_{\mathrm{ref}}-G_{\sigma }\left(I_{\mathrm{ref}}\right)\right)\right|}^{2},$$

where F(·) is the two-dimensional Fourier transform, and $G_{\sigma }\left(\cdot \right)$ denotes Gaussian-like smoothing. With $\rho \left(f_{x},f_{y}\right)=\sqrt{f_{x}^{2}+f_{y}^{2}}$, the spectral center and bandwidth are computed as

(16) $$\begin{array}{cc} f_{c} & =\frac{\sum_{f_{x},f_{y}}\rho \left(f_{x},f_{y}\right)P\left(f_{x},f_{y}\right)}{\sum_{f_{x},f_{y}}P\left(f_{x},f_{y}\right)}, \end{array}$$

(17) $$\begin{array}{cc} b & =\sqrt{\frac{\sum_{f_{x},f_{y}}{\left(\rho \left(f_{x},f_{y}\right)-f_{c}\right)}^{2}P\left(f_{x},f_{y}\right)}{\sum_{f_{x},f_{y}}P\left(f_{x},f_{y}\right)}}. \end{array}$$

Band-limited random fields are then synthesized in the Fourier domain according to $f_{c}$ and *b*. The resulting style field $S_{i}$ combines the reference gray-level range, the vertical energy envelope, a low-frequency random field, and a band-limited texture field. Therefore, the generated B-scans inherit realistic intensity and spectral statistics but do not inherit the spatial details of $I_{\mathrm{ref}}$.

The method is described below from three aspects: physics-constrained component construction, transformer modulation and fusion, and noisy B-scan synthesis with reference-similarity suppression.

### 3.1. Physics-Constrained Component Construction

The clean target response is generated by sampling one or more hyperbolic reflection events. For the *k*-th target, the range trajectory is modeled as

(18) $$y_{k}\left(x\right)=d_{k}+\kappa_{k}{\left(x-x_{k}\right)}^{2},$$

where $d_{k}$ is the normalized target depth, $x_{k}$ is the lateral target position, and $\kappa_{k}$ controls the curvature related to the propagation velocity and target depth. The target echo is synthesized as a damped bipolar waveform around $y_{k}\left(x\right)$, which approximates the oscillatory response of a GPR pulse. Multiple targets with different amplitudes, polarities, widths, and lateral apertures can be sampled in one B-scan. The target mask is obtained by thresholding the absolute clean response and applying local dilation, so weak target edges are retained.

The direct-coupling component follows the physical observation that antenna leakage appears as an early, coherent, nearly horizontal band. Its center is initialized from the strongest early-time row of the reference image and then randomly perturbed. Several positive and negative ringing lobes are added around this center

(19) $$C_{i}^{\mathrm{dc}}\left(x,y\right)=r_{i}\left(x\right)\sum_{\ell }a_{\ell }\exp\left[-\frac{{\left(y-y_{i}^{\mathrm{dc}}-\Delta_{\ell }\right)}^{2}}{2\sigma_{\ell }^{2}}\right],$$

where $r_{i}\left(x\right)$ is a lateral ripple term, $y_{i}^{\mathrm{dc}}$ is the sampled direct-coupling time gate, and $\Delta_{\ell }$ represents the offsets of different ringing lobes.

The stochastic background component combines dense receiver noise, row-correlated background fluctuation, band-limited interference, random time-gate interference, and sparse impulsive scan columns. Receiver-noise and band-limited terms represent noise or acquisition interference, whereas row-correlated and scan-column patterns are structured background disturbances. They are grouped as a composite augmentation field and are not all classified as geological clutter. The direct-coupling component and the multipath component represent structured clutter. The multipath component is generated from two mechanisms. First, several wavy horizontal interface reflections are sampled to represent repeated reflections at underground layer boundaries. Second, delayed virtual target responses are produced by shifting and broadening the direct target hyperbola, which approximates the transmitting-side and receiving-side multipath described by the underground clutter model.

### 3.2. Transformer Modulation and Fusion

The generator contains three transformer branches, corresponding to direct coupling, the stochastic background noise/interference field, and multipath clutter. Each branch takes the reference-statistical style field and one physical component as a two-channel input. A convolutional stem extracts local features, patch embedding converts the feature map into tokens, and a transformer encoder captures long-range dependencies along the scan and range directions. The three branches use different patch shapes to match the spatial properties of the non-target components: 16×64 patches for direct coupling, 16×32 patches for the stochastic background field, and 32×32 patches for multipath. The output of the *q*-th branch is

(20) $$B_{i}^{q}=\tanh\left[T_{q}\left(\left[S_{i},\;C_{i}^{q}\right]\right)\right],\;q\in \left\{\mathrm{dc},\mathrm{rn},\mathrm{mp}\right\},$$

where $T_{q}\left(\cdot \right)$ denotes the transformer modulation branch. The modulated clutter component is then obtained as

(21) $${\tilde{C}}_{i}^{q}=C_{i}^{q}\left(1+\gamma_{q}B_{i}^{q}\right),$$

where $\gamma_{q}$ is a bounded modulation coefficient. A convolutional fusion head further combines the style field, clean target response, and three modulated clutter components

(22) $$F_{i}=\tanh\left[\Phi \left(\left[S_{i},\;X_{i},\;{\tilde{C}}_{i}^{\mathrm{dc}},\;{\tilde{C}}_{i}^{\mathrm{rn}},\;{\tilde{C}}_{i}^{\mathrm{mp}}\right]\right)\right],$$

where Φ(·) is the fusion network. This structure allows the transformer to adjust physically generated clutter fields while preserving the interpretability of each component.

For reproducibility, the generator instantiates the three transformer modulation branches and the convolutional fusion head in PyTorch and uses them in an inference-only forward-synthesis procedure. The direct-coupling branch uses a 16×64 patch size, embedding dimension 72, two encoder layers, four attention heads, a local convolution kernel of 5, and dropout 0.06. The stochastic-background branch uses a 16×32 patch size, embedding dimension 72, two encoder layers, four attention heads, a local kernel of 3, and dropout 0.16. The multipath branch uses a 32×32 patch size, embedding dimension 96, three encoder layers, four attention heads, a local kernel of 5, and dropout 0.08. The fusion head receives five channels, namely the style field, clean target response, and the three modulated clutter maps, and applies convolutional layers with 48 hidden channels before producing one output channel.

The transformer branches and fusion head are not pre-trained and are not optimized with a separate training dataset in the current implementation. Specifically, the code constructs the modules, applies the declared parameter initializations, switches the complete generator to evaluation mode with model.eval(), and generates samples inside a torch.no_grad() context. There is no training data loader, target batch, loss or criterion, optimizer, back-propagation operation, epoch/iteration loop, or externally loaded trained checkpoint in generate_bscan_dataset.py; therefore, the training data, loss function, optimizer, learning rate, and number of training epochs are not applicable to this generator. The file generator_model.pt records the instantiated module state and generation metadata, rather than a trained model checkpoint.

The transformer is retained as a core architectural innovation of the proposed framework, but its role in the present training-free implementation must be distinguished from supervised representation learning. Here, self-attention acts as an input-dependent random-feature operator that introduces nonlocal interactions between the reference-statistical style field and each physically generated clutter component. The bounded factors $\gamma_{q}B_{i}^{q}$ restrict this modulation around the corresponding physical field, so direct coupling, stochastic background, and multipath remain separately identifiable. Thus, the transformer provides a component-specific global modulation pathway that is absent from the physics-only synthesis, whereas the physical model supplies the target geometry and clutter semantics. To isolate this contribution, we implemented three matched configurations: a physics-only variant that sets the transformer and fusion residuals to zero, a fusion-only variant that bypasses the three transformer branches, and the complete model. The complete model was additionally evaluated with five independent neural initialization seeds while every configuration received exactly the same sampled physical components.

The only image supplied to the generator is the reference B-scan, which is used for statistical conditioning rather than parameter learning or pixel-level copying. Its grayscale mean and standard deviation, vertical energy profile, and spectral moments condition the sampled style field; the target, direct-coupling, stochastic-background, and multipath maps are then generated from the physical and stochastic rules described above. Randomized target and clutter parameters are sampled for each batch, and the random streams are controlled by the declared seed to make a run repeatable. Thus, the transformer modules modulate physically generated components during forward synthesis, while the physical maps and fixed configuration gains provide the effective constraints and supervision products. This distinction clarifies that the proposed method is a procedural, constraint-guided dataset generator, not a separately trained black-box image-generation network.

### 3.3. Noisy B-Scan Synthesis and Reference-Similarity Suppression

The final B-scan is synthesized from normalized residuals of the style field, target response, modulated clutter components, transformer textures, and fusion output. For a component *U*, its local residual is defined as

(23) $$H_{k}\left(U\right)=\mathrm{Norm}\left(U-A_{k}\left(U\right)\right),$$

where $A_{k}\left(\cdot \right)$ is average smoothing with kernel size *k*, and Norm(·) denotes zero-mean amplitude normalization. The preliminary B-scan is

(24) $$\begin{array}{cc} Y_{i}^{0}= & \alpha_{s}H_{13}\left(S_{i}\right)+\alpha_{t}H_{19}\left(X_{i}\right)+\alpha_{d}H_{17}\left({\tilde{C}}_{i}^{\mathrm{dc}}\right)\\ \; & +\alpha_{r}H_{7}\left({\tilde{C}}_{i}^{\mathrm{rn}}\right)+\alpha_{m}H_{25}\left({\tilde{C}}_{i}^{\mathrm{mp}}\right)+\alpha_{f}H_{9}\left(F_{i}\right). \end{array}$$

To prevent the generated image from becoming too similar to the reference B-scan, the projection of $Y_{i}^{0}$ onto the high-frequency residual of the reference image is suppressed

(25) $$Y_{i}^{1}=Y_{i}^{0}-\lambda \frac{\langle Y_{i}^{0},R_{\mathrm{ref}}\rangle }{\langle R_{\mathrm{ref}},R_{\mathrm{ref}}\rangle }R_{\mathrm{ref}},\;R_{\mathrm{ref}}=H_{17}\left(I_{\mathrm{ref}}\right),$$

where λ is the reference-suppression coefficient. Finally, $Y_{i}^{1}$ is rescaled to the gray-level range estimated from $I_{\mathrm{ref}}$ and clipped to [0,1]. This step maintains realistic contrast and frequency distribution while explicitly discouraging pixel-level copying of the reference image.

The smoothing kernels in Equation (24) are fixed engineering hyperparameters rather than learned quantities. All kernels are odd, so stride-one average filtering preserves the center alignment of the residual. The selected scales were matched to the characteristic spatial extent of each component after the B-scan is resized to 512×512. The larger windows $H_{25}$ and $H_{19}$ are used for the clean target and multipath terms because these responses occupy broader hyperbolic envelopes and delayed layer-like structures. The intermediate windows $H_{17}$ and $H_{13}$ are used for direct coupling and the style field because these terms contain coherent scan-direction bands whose envelope should be preserved, but whose local oscillation should be suppressed. The smaller windows $H_{7}$ and $H_{9}$ are reserved for stochastic background and fusion texture because these terms contain fine local fluctuations that would be over-smoothed by a larger kernel. Thus, the kernel sizes are selected from the expected spatial scale of the corresponding physical or learned component: a larger window extracts a broader residual structure, whereas a smaller window retains finer interference details. The fixed odd-valued set provides a reproducible compromise between preserving target/clutter morphology and removing slowly varying background content.

The coefficients $\alpha_{s}$, $\alpha_{t}$, $\alpha_{d}$, $\alpha_{r}$, $\alpha_{m}$, and $\alpha_{f}$ are manually specified fixed hyperparameters rather than learned network parameters. Equation (24) is not optimized in the present study. Instead, the implementation assigns fixed weights to balance the contributions of the target and the three clutter components after each residual has been independently normalized. Specifically, the effective target, direct-coupling, stochastic-background, and multipath weights are 0.30×0.58=0.174, 0.22×0.66=0.1452, 0.18×0.36=0.0648, and 0.26×0.58=0.1508, respectively. The style residual uses 0.30×0.55=0.165, with an additional reference-statistical texture term weighted by 0.10×0.55=0.055; the transformer-texture and fusion residuals use fixed weights of 0.050 and 0.070. These values give the target and structured clutter terms larger contributions than the fine stochastic-background term, while retaining style and fusion details without allowing them to dominate the composite B-scan. The fixed gains also keep the synthesized image within a realistic contrast range after rescaling by the reference mean and standard deviation. The coefficients can be re-tuned for a different soil or target scenario, but they are not learned from data in this work.

Although Equation (24) currently uses six independently specified gains, their number can be reduced in future implementations by exploiting the physical roles of the normalized components. Specifically, the target response can form one group, the style, direct-coupling, and multipath terms can form a structured-background group, and the stochastic-background and fusion terms can form a fine-texture group. Fixed intra-group ratios may be determined once from the normalized component energies, leaving only three group-level gains to be calibrated. Imposing a sum-to-one constraint on these gains would further reduce the formulation to two independent degrees of freedom. The remaining coefficients could then be selected automatically on a small validation set using target-preservation, radar-statistical consistency, and clutter-spectrum criteria. This grouped formulation would reduce manual tuning while retaining the physical interpretability and inference efficiency of the present generator. The current experiments retain the six fixed gains reported above; evaluation of the reduced parameterization across different sites and frequency bands is left for future work.

### 3.4. Physical-Consistency Evaluation Protocol

To quantify the accuracy sacrificed by the simplified component model, five existing gprMax/FDTD B-scans were selected: M1T7S1, M2T5S1, M4T2S1, M5T10S1, and M6T6S1. Together, they cover five target-path dielectric profiles ($\epsilon_{r}=6$, 9, 16, 27, and a 9/16 layered path): homogeneous, two-layer media, three-layer media, and free-space, PVC, PEC sphere, cylinder, and box targets. All scenes use a 1 GHz impulse, a 50 ns time window, 0.005 m FDTD cells, 0.03 m scan steps, and a 0.40 m transmitter–receiver separation. The corresponding material, layer, antenna, and target parameters were parsed directly from the gprMax input files.

Raw $E_{z}$ fields were exported without display normalization. For both the generated and FDTD B-scans, the coherent background was estimated from traces at least 0.85 m from the surveyed target, and arrival time was referenced to the observed direct wave to remove source-launch offsets. The target trajectory was picked from the analytic-signal envelope inside a geometry-predicted time gate and fitted robustly as $t\left(x\right)=ax^{2}+bx+c$, where *a* is the reported hyperbola curvature. Amplitude was evaluated as the target-envelope peak relative to the direct-wave peak in decibels. Multipath delay and the multipath-to-primary envelope ratio were extracted from the strongest significant delayed peak. The scene-conditioned analytical realization retained the proposed model’s fixed target/direct gains and was not fitted to the evaluated FDTD features. The available measured B-scan was checked only in normalized image coordinates because the PNG does not retain its time interval, trace spacing, antenna wavelet, soil profile, target survey, or linear-amplitude calibration; it was therefore not pooled with the physical-unit FDTD errors.

The implementation writes the generated dataset as a compressed npz file containing noisy images, clean target responses, target masks, separated clutter components, transformer modulation maps, similarity diagnostics, and metadata. Preview images are also saved for visual inspection. The similarity diagnostics include the mean squared error and correlation between generated images and the reference image. These values are used only as quality control indicators; the objective is not to minimize the difference from the reference but to maintain realistic GPR statistics while preserving physical diversity. Because all component sampling, transformer modulation, and fusion operations are tensorized, the generator can produce large denoising datasets efficiently on a GPU.

## 4. Results

The experimental evaluation was designed to verify whether the proposed clutter-constrained transformer generator can rapidly construct physically interpretable GPR denoising data while avoiding direct copying of the reference B-scan. All experiments were conducted on a laptop workstation running Windows 11 (build 26200.8655), equipped with an Intel(R) Core(TM) Ultra 9 185H CPU with 22 logical processors, 31.5 GiB of system memory, and an NVIDIA GeForce RTX 4050 Laptop GPU with 6141 MiB of video memory. The implementation used Python 3.12.13 and PyTorch 2.9.0 with CUDA 12.8 support. The evaluation included field-scene documentation, batch data generation and export-integrity verification, comparison with a gprMax FDTD B-scan, frequency-dependent noisy B-scan synthesis, statistical diagnostics of generated samples, output-level comparison with representative GAN-, diffusion-, and forward-model-based GPR generation methods, and downstream denoising comparison on measured data. The downstream measured-data experiment used a 1200-sample GPR dataset. The 100-sample count reported elsewhere refers only to the controlled synthetic generator run used for throughput, output-integrity, and component-diagnostic measurements; it does not describe the size of the measured dataset used for the denoising result.

**Figure 4:**
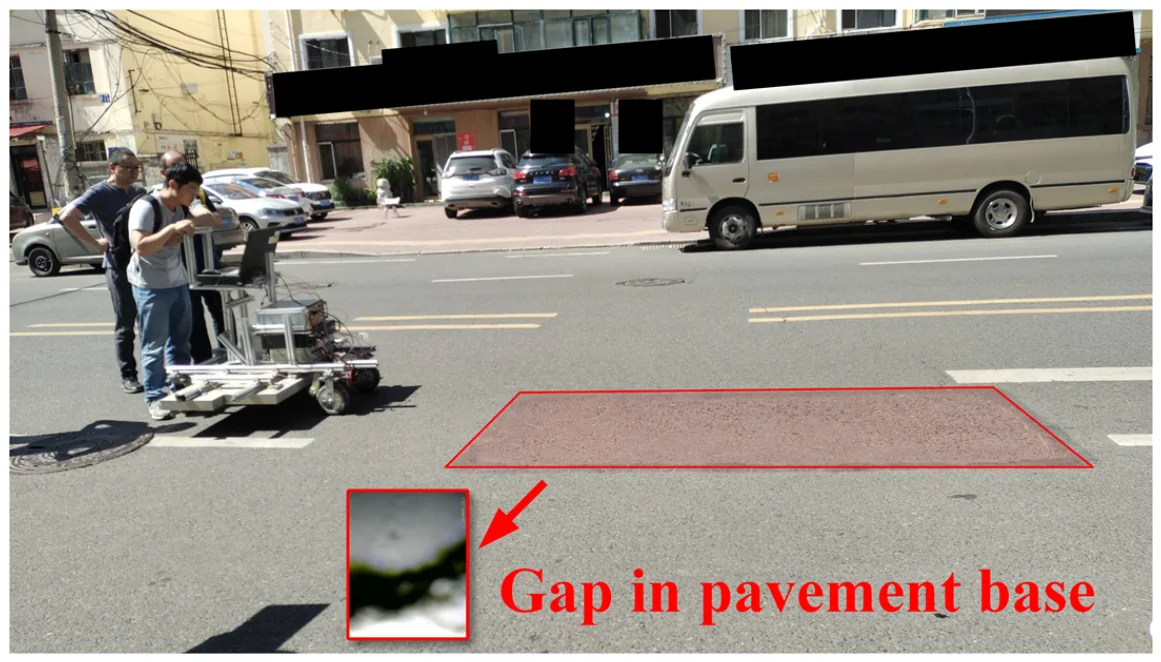
Field GPR survey on an urban road pavement. The cart-mounted GPR system scans the marked pavement region, while the red arrow and inset identify a gap in the pavement base.

To connect the synthetic results with the physical acquisition problem, a field survey scene is shown in Figure 4. The experiment was conducted on an urban road pavement using a cart-mounted GPR system. The red polygon marks the investigated pavement region, while the inset image and red arrow identify a gap in the pavement base. This field scene demonstrates that practical GPR measurements contain responses from pavement-layer interfaces and structural defects together with acquisition and environmental clutter. These observations motivate the use of physically interpretable target and clutter components when constructing synthetic denoising data.

### 4.1. Batch Generation and Data Integrity

The proposed generator was evaluated by producing 100 GPR B-scan samples with a spatial size of 512×512 pixels. The run used a batch size of 10 and was executed on a CUDA-enabled GPU. The run log recorded a total generation time of 38.36 s, corresponding to 2.61 samples/s and 0.384 s per generated B-scan. Since each sample contains one noisy image and five supervision or component maps, the experiment produced 600 primary maps, including 500 target or clutter supervision maps. This corresponds to 157.3 million stored pixel values, of which 131.1 million belonged to the target, clean-response, or clutter-component outputs. The effective output rate was therefore 15.64 maps/s, or 4.10 million stored pixel values/s. The exported dataset was then reopened from the compressed npz file, and all statistics were recomputed from the stored arrays to verify the consistency of the comparison report. The maximum absolute deviation between the stored and recomputed diagnostics was $1.3\times 10^{-9}$ for MSE and $1.3\times 10^{-8}$ for correlation.

Table 2 shows that the proposed method does not generate image-level B-scans only. Each sample is accompanied by a target mask, a clean target response, and three physically interpretable clutter components. This structure directly supports supervised denoising, component-wise ablation, and quantitative clutter suppression analysis.

**Figure 5:**
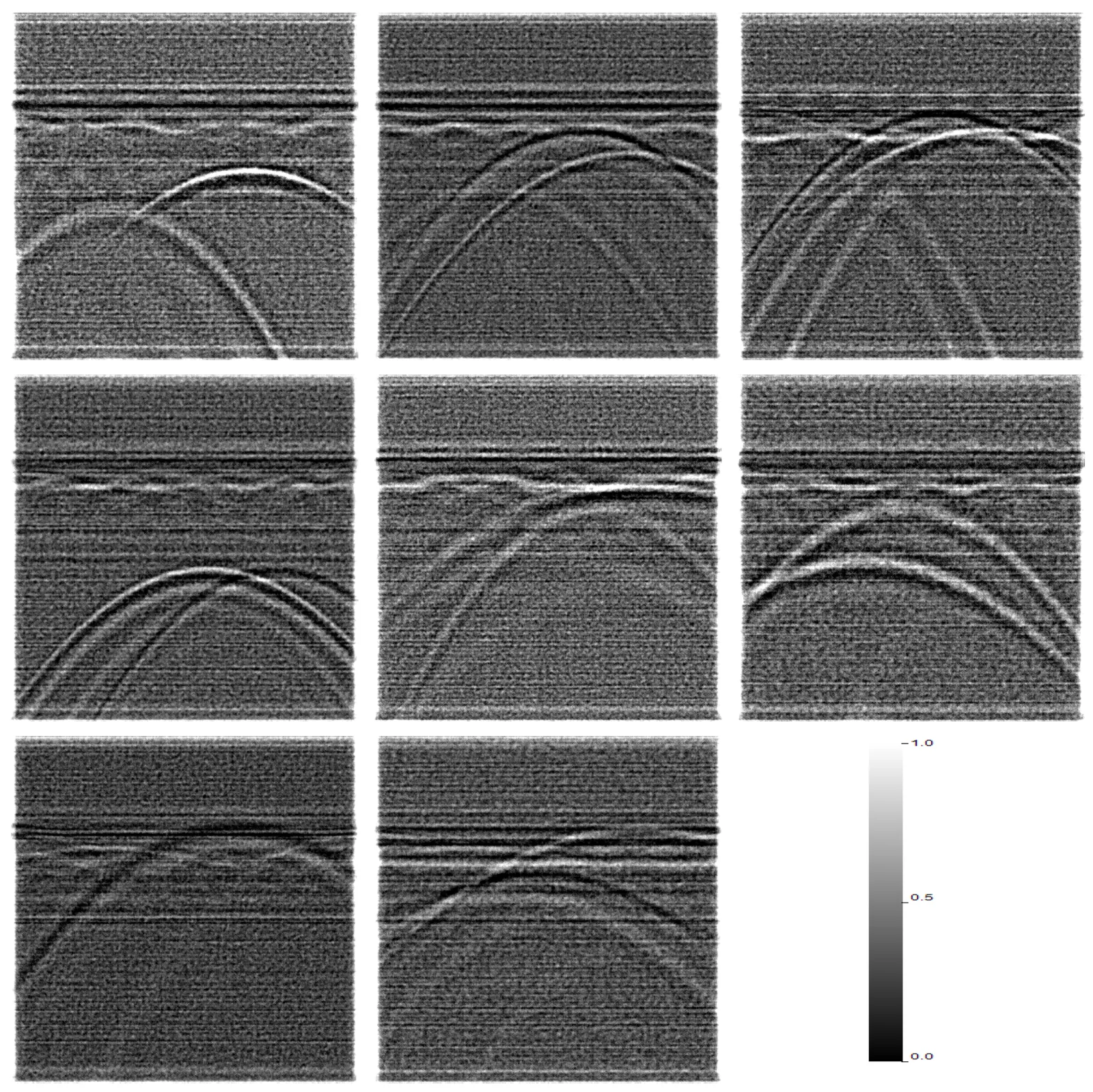
Representative contrast-enhanced noisy GPR B-scan previews generated by the proposed method. The eight examples were selected from the exported 100-sample dataset and displayed with a common percentile-based grayscale mapping to improve visibility of weak responses.

Figure 5 provides a qualitative view of the generated dataset. The samples contain shallow coherent bands, scan-direction background fluctuations, localized random interference, and hyperbola-like target responses with different positions, depths, apertures, and amplitudes. These visual characteristics are consistent with the data products in Table 2: the noisy B-scan is not an isolated image, but the composite observation corresponding to an exported clean response, target mask, direct-coupling field, stochastic-background field, and multipath field. Therefore, the generated dataset can be used as paired training data in which the denoising model is exposed to clutter-rich B-scans while the desired target-preserving output and component-level supervision remain available. The visual variation among the eight previews also supports the diagnostic objective of avoiding a simple perturbation of one reference image; target geometry and clutter layout vary across samples while the global grayscale style remains controlled.

**Figure 6:**
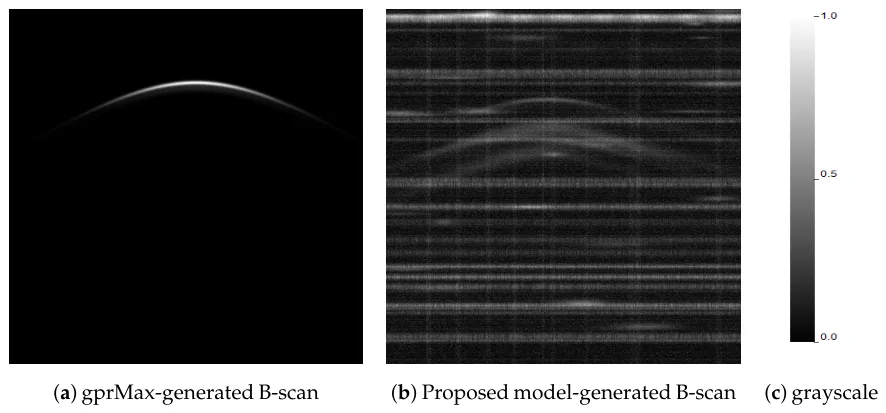
Comparison between the gprMax-generated B-scan and the B-scan generated by the proposed model. Compared with the cleaner gprMax target response, the proposed model produces richer noise and clutter details while retaining the main hyperbola-like target structure.

To compare the proposed generator with the conventional electromagnetic forward-modeling result, Figure 6 shows a gprMax-generated B-scan and a B-scan generated by the proposed model. The gprMax result mainly presents a clear target hyperbola with a relatively simple background. In contrast, the proposed model preserves the target-related hyperbola while introducing richer clutter details, including shallow coherent bands, laterally varying background texture, horizontal layer-like interference, and weak vertical trace disturbances. This comparison indicates that, for denoising-dataset construction, the proposed model can provide more complex noisy observations than a clean forward-modeled target response, which helps expose the denoising network to more diverse clutter patterns.

**Figure 7:**
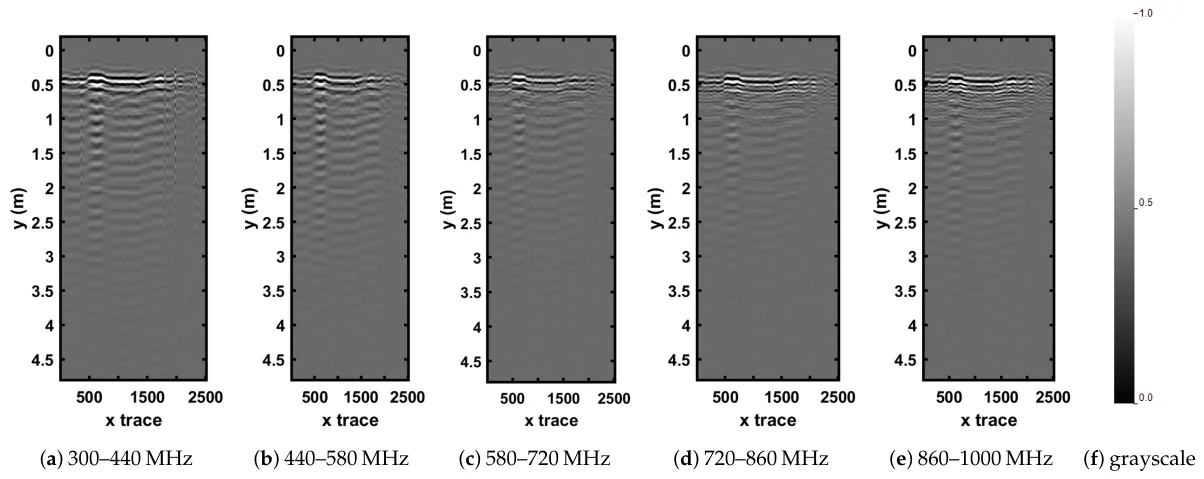
Contrast-enhanced generated B-scan comparison at different frequency bands. The five panels correspond to 300–440, 440–580, 580–720, 720–860, and 860–1000 MHz, respectively, using a common percentile-based grayscale mapping.

Frequency variability was further evaluated by comparing generated noisy B-scans at five frequency bands. The numbers in the file names indicate the corresponding frequency ranges in MHz. To keep the comparison focused on frequency-dependent clutter behavior, the five images in Figure 7 are arranged from low to high frequency.

Figure 7 shows that noise and clutter morphology vary clearly with frequency. In the 300–440 MHz band, the clutter field is dominated by wider low-frequency oscillations and longer vertical ringing, and the target-related response is accompanied by broader deep-range fluctuation. As the frequency increases to 440–580 and 580–720 MHz, the shallow layer responses become more compact, and the horizontal interference around the near-surface interface becomes clearer. In the 720–860 and 860–1000 MHz bands, fine-scale texture and shallow high-frequency clutter are more prominent, while the deep background becomes weaker and smoother because higher-frequency components attenuate more rapidly in lossy underground media. These observations are consistent with GPR physics: lower frequencies provide stronger penetration but lower resolution, whereas higher frequencies improve shallow detail resolution but are more sensitive to attenuation and local interface roughness. Therefore, the proposed generator can represent frequency-dependent noise characteristics and provide denoising samples with different clutter spectra.

### 4.2. Statistical Diagnostics of Generated Samples

The numerical diagnostics of the 100 generated samples are listed in Table 3. The similarity values were computed against the reference B-scan that was used only to estimate gray-level range, vertical energy distribution, and spectral statistics. The mean correlation to the reference was −0.0210, and the maximum correlation among all generated samples was only 0.0779. These values indicate that the generated samples do not form pixel-level copies of the reference image. At the same time, the normalized mean squared error to the reference remained within a narrow range from 0.01216 to 0.01325, which indicates that the overall gray-level scale and statistical appearance remained controlled.

The target-mask coverage was concentrated between 10.08% and 13.84%, with a mean of 11.74%. Therefore, the generated masks were neither empty nor dominated by target pixels, which is important for stable supervised denoising. The target, direct-coupling, stochastic-background, and multipath-clutter energy values were all non-zero across the dataset. The stochastic-background energy was relatively stable, with a standard deviation of 0.0220, while the target and multipath energies varied more strongly. This behavior is consistent with the design objective: background interference should remain statistically realistic, whereas target depth, target strength, and delayed multipath should vary across samples.

### 4.3. Ablation of Transformer Modulation and Initialization Stability

A matched ablation was conducted to determine the contribution of the untrained transformer branches independently of physical-scene randomness. One hundred 512×512 B-scans were evaluated under three configurations: physics only, with all neural residuals disabled; fusion only, with the convolutional fusion head retained but the transformer branches bypassed; and the complete transformer–fusion model. For the complete model, five independent neural initialization seeds (101, 202, 303, 404, and 505) were tested. Each batch of target, direct-coupling, stochastic-background, multipath, and style components was sampled only once and passed unchanged to every configuration and initialization. All images were resized to 256×256 and evaluated in one common feature space. RFID, RIS, and R-LPIPS denote the radar-domain diagnostic counterparts defined by the common offline protocol rather than standard ImageNet-based perceptual metrics.

Table 4 shows that the complete model changed the output in a measurable but bounded manner. Relative to the physics-only control, the five-seed mean RFID decreased by 0.80%, and RIS increased by 4.36%. However, R-LPIPS increased by 0.21%, and pairwise MS-SSIM increased by 1.16%, so the untrained transformer did not improve every diagnostic consistently. The clean-target correlation changed from 0.1760 for the physics-only output to 0.1566±0.0116 for the complete model, while target/background contrast changed from 0.002931 to 0.002727±0.000051. The complete and physics-only images remained close, with a paired MSE of 0.000024±0.000008 and paired MS-SSIM of 0.9944±0.0016. Across pairs of complete-model initializations, the matched-image MSE was 0.000048±0.000016, and MS-SSIM was 0.9888±0.0032. These mixed metric directions and seed-dependent variations show that the current untrained transformer should not be interpreted as having learned the GPR distribution. Its demonstrated role is a small, bounded, component-specific nonlocal modulation of the physical generator. We retain this transformer pathway as the central architectural mechanism for coupling global context to separately interpretable clutter components, while recognizing that training or deterministic calibration is required to obtain initialization-independent gains.

### 4.4. Quantitative Validation Against gprMax/FDTD

Table 5 shows that the proposed simplified model preserves first-order propagation geometry more reliably than calibrated scattering strength or interface-dependent multipath. The direct-referenced target-apex delay has an MAE of 0.725 ns and an MAPE of 4.70%, while the fitted hyperbola curvature has an MAE of 1.665 ns $m^{-2}$ and an MAPE of 14.41%. These results support the use of the generated data for preserving target arrival and hyperbola-like morphology. In contrast, the target/direct amplitude error is 44.50 dB, the multipath-delay MAPE is 45.53%, and the multipath/primary amplitude error is 4.40 dB. The large target/direct error is consistent with the deliberate target-enhancing fixed gains used for denoising supervision, whereas the multipath error reflects the use of a small number of fixed delayed replicas instead of a full interface-dependent wave solver. Therefore, the quantitative evidence supports a fidelity–efficiency trade-off: the method retains the principal target geometry and rapidly exports six component-labeled maps per B-scan, but it sacrifices calibrated amplitude and detailed multipath accuracy. The transformer remains the core architectural mechanism for component-specific nonlocal modulation; this comparison evaluates the accuracy of the physical component model and does not replace the transformer ablation in Table 4.

### 4.5. Quantitative Comparison with Reproducible Baselines

The proposed generator is compared with four representative GPR data generation models: GPR-GAN [9], GAN-based B-scan augmentation with multiscale discrimination [10], GPR-VMD-Diffusion [15], and FM-GAN [13]. To ensure a fair and rigorous comparison, all methods were reimplemented and evaluated under the same dataset, hardware, and evaluation protocol. Specifically, all methods were trained or configured on the same training split, generated 100 B-scans of size 512×512, and were evaluated using the same radar-domain metrics on the same test set. The FDTD-plus-stochastic-noise baseline was also evaluated under this identical protocol. No method-specific tuning or dataset-specific adaptation was allowed after the protocol was fixed.

**Figure 8:**
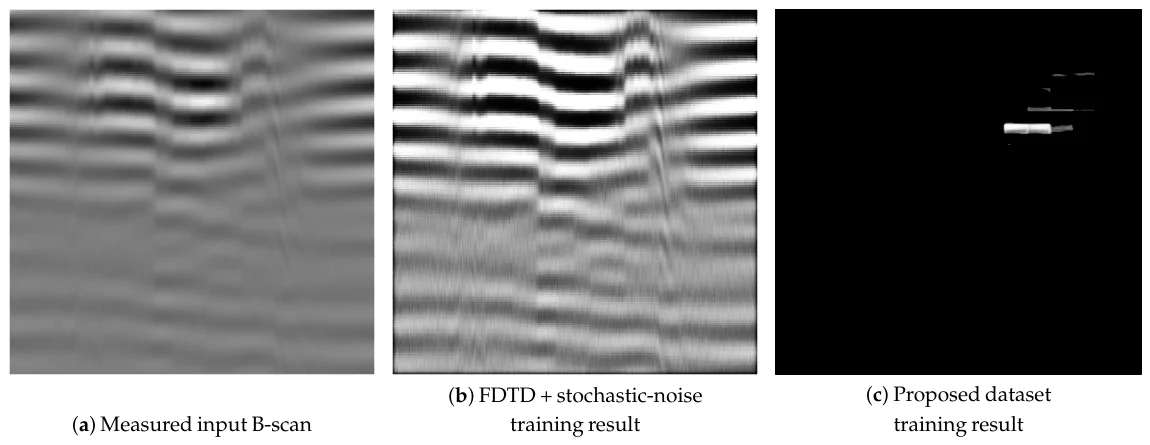
Comparison of denoising results obtained from two transformer-based networks. The input grayscale B-scan is shown in (**a**). The network trained on the FDTD dataset with added stochastic acquisition noise is shown in (**b**), and the network trained on the proposed dataset is shown in (**c**).

All images were converted to grayscale, normalized with the same global percentile range, and resized to 256 × 256 for metric computation. Since the workspace does not contain pretrained ImageNet Inception or LPIPS weights, the reported values are explicitly defined as radar-domain counterparts: RFID is a Frechet distance in a deterministic feature space containing intensity statistics, scan-direction profiles, gradients, and Fourier-spectrum descriptors; RIS is an Inception-score-style value obtained from soft assignments to eight unsupervised radar-feature prototypes; pairwise MS-SSIM measures within-set structural similarity, where a lower value indicates greater pixel-level diversity; and R-LPIPS is a multiscale radar-texture feature distance to the nearest measured/reference B-scan. The reference set consists of the reference B-scan examples used for metric calculation and the measured input B-scan shown in Figure 8a.

Table 6 provides a strict identical-protocol comparison. The proposed model achieves the lowest RFID (1703.823) and R-LPIPS (1.293) among all evaluated methods, indicating that its generated samples are closest to the measured/reference B-scan distribution in both global radar statistics and multiscale texture features. The proposed model also achieves the highest RIS (1.286), showing a broader occupancy of the radar-feature prototypes. Compared with the best representative baseline, GPR-VMD-Diffusion, the proposed model reduces RFID by 7.65%, increases RIS by 4.55%, reduces pairwise MS-SSIM by 2.79%, and reduces R-LPIPS by 1.30%. Relative to the FDTD-plus-stochastic-noise baseline, the proposed dataset reduces RFID by 16.79% and R-LPIPS by 4.96% and increases RIS by 22.43%. Pairwise MS-SSIM is higher for the proposed set (0.7145 versus 0.2196 for FDTD + stochastic acquisition noise). Because lower pairwise MS-SSIM denotes greater pixel-level diversity, the FDTD-plus-stochastic-noise set has more unstructured pixel variation; however, this variation is dominated by random perturbations. The proposed set deliberately maintains a more coherent physical style while varying target geometry, depth, direct coupling, stochastic background, and multipath components. Overall, the strict identical-protocol results demonstrate that the proposed method provides a better realism-controlled-diversity balance for denoising-data construction.

## 5. Discussion

The experimental results support the working hypothesis that a transformer generator constrained by an underground clutter physical model can provide fast and interpretable GPR denoising data. The measured throughput of 2.61 full-resolution B-scans per second, equivalent to 15.64 exported maps per second, demonstrates that the proposed tensorized generator is suitable for batch dataset construction. More importantly, the output is not limited to visually plausible B-scans. Each generated sample provides six directly usable maps, including five supervision or component maps, which are difficult to obtain from field data and are not directly provided by general image-level GAN or diffusion generation methods.

The FDTD comparison qualifies this efficiency claim by identifying the physical information retained and discarded by the acceleration. The low propagation-delay error and moderate curvature error show that the analytical target path preserves the timing and hyperbolic geometry most relevant to target-aware denoising. The substantially larger calibrated-amplitude and multipath-delay errors show that this efficiency is achieved partly by replacing full-wave scattering, attenuation, and repeated interface interactions with fixed-gain components and a small set of delayed replicas. Accordingly, the reported throughput should be interpreted as the efficiency of constructing component-labeled training data, not as evidence that the generated B-scans are full-wave-equivalent. A numerical acceleration factor relative to gprMax is not claimed because the two pipelines were not timed under an identical end-to-end protocol.

The reference-similarity diagnostics also show that the generator balances realism and diversity. The reference image contributes only global gray-level, vertical-energy, and spectral statistics, while randomized target, medium, direct-coupling, stochastic-background, and multipath parameters determine the sample-specific structure. The near-zero mean correlation to the reference indicates that the generated B-scans are not obtained by simple template copying. Meanwhile, the bounded MSE range indicates that the generated images remain within a controlled statistical domain. This behavior is important for denoising because excessive similarity would reduce dataset diversity, whereas excessive deviation from realistic B-scan statistics would weaken the transferability of the trained denoising model.

The generated dataset should therefore be interpreted as a component-labeled training corpus rather than only as image augmentation. The preview images in Figure 5 show that each noisy B-scan contains multiple interference patterns that a denoising model would encounter in practical GPR processing, while the exported arrays provide the corresponding clean target response and clutter decomposition. This pairing allows the same dataset to support several training objectives, including direct noisy-to-clean regression, target-mask-aware preservation, direct-coupling suppression, stochastic-background suppression, and multipath-specific ablation. Since the current 100-sample run already contains 600 primary maps and 157.3 million stored pixel values, the generator can be scaled to larger synthetic corpora without requiring manual annotation of field B-scans. In practical applications, the generated component-labeled data can be combined with measured data so that the training set retains dense supervision while adapting to site-specific acquisition effects.

The current experiment demonstrates that the generator can export a structured 100-sample corpus efficiently, but it does not by itself quantify robustness across sites or acquisition conditions. A complete generalization study should use stratified sampling over relative permittivity or moisture-related effective parameters, antenna frequency, target depth and geometry, and clutter strength. The synthetic data should be divided by condition rather than by randomly shuffled images, with at least one held-out combination of medium, frequency, and target configuration. The denoising model should then be evaluated on independent measured B-scans from multiple sites or survey lines, with one site kept completely outside training and model selection. Results should be reported per site and as an aggregate mean with standard deviation, using target-preservation measures, clutter-suppression measures, and PSNR/SSIM only when clean references are available.

The existing simulations provide partial condition-coverage evidence that is relevant to, but does not replace, the requested cross-condition experiment. The five matched gprMax/FDTD scenes in Table 5 span relative-permittivity paths of 6, 9, 16, 27, and 9/16, together with homogeneous, layered, and multilayer propagation and sphere, cylinder, and box targets. In addition, Figure 7 shows five frequency bands from 300–440 to 860–1000 MHz, and Table 7 reports model-based SNR/SCR estimates associated with the three selected test areas. These results demonstrate that the generator can represent changes in propagation geometry, target morphology, frequency-dependent texture, and moisture-related background separation. They do not establish site-independent denoising robustness because the FDTD scenes are controlled simulations, the frequency sweep is qualitative, the moisture values are coupled to different test areas, and the current corpus contains only 100 generated samples. A definitive Comment 5 evaluation still requires a larger stratified synthetic corpus and measured B-scans from independent areas with an area-held-out protocol.

Figure 8 compares the measured input and the two transformer denoising outputs. Visually, the network trained on the FDTD dataset with stochastic-noise augmentation in Figure 8b still retains a large amount of horizontal striping and broad background energy, whereas the network trained on the proposed dataset in Figure 8c suppresses most diffuse clutter and leaves only sparse, localized reflection traces. To quantify this difference, several image-level diagnostics were computed on the displayed 512×512 PNG results. Relative to the measured input in Figure 8a, the FDTD-noise output increases the mean gray value from 122.255 to 143.464 and the RMS value from 125.456 to 156.869, while the proportion of pixels above 128 increases from 29.144% to 62.013%. Its average gradient magnitude also grows from 4.033 to 15.920, which indicates that high-frequency striping survives the filtering stage. By contrast, the proposed-data output reduces the mean gray value to 0.922 and the RMS value to 12.765, and it suppresses the proportions of pixels above 20 and 128 to 0.625% and 0.286%, respectively. The average gradient magnitude drops to 0.303, showing that the residual background is much smoother and far sparser. Compared with the FDTD-noise output, the proposed-data output reduces the mean intensity by 99.36%, the RMS intensity by 91.86%, the area ratio of pixels above 128 by 99.54%, and the average gradient magnitude by 98.10%. These values indicate that the proposed dataset provides a more effective training prior for clutter suppression and target preservation than the FDTD-noise dataset because it encourages the transformer to remove diffuse interference instead of merely rebalancing the intensity distribution.

Relation to the physical problem. The synthesis process changes with the ground profile because dielectric contrast, interface reflection, propagation velocity, and attenuation alter both the direct target path and the delayed multipath paths. It changes with the target because target material, reflection polarity, depth, curvature, lateral aperture, and effective size control the strength and shape of the hyperbolic response. For a fixed scene under different excitation frequencies, the expected changes are frequency-dependent resolution, penetration, bandwidth, ringing, and clutter spectrum. Figure 7 provides a qualitative five-band comparison of this effect, showing the transition from broader low-frequency ringing to finer shallow high-frequency clutter. Therefore, the proposed synthesis process is physically interpretable at the component level: ground parameters affect propagation and clutter structure, target parameters affect the diffraction signature, and excitation frequency affects resolution, penetration, and spectral distribution.

Table 7 uses the moisture contents of the three selected test areas: 1% for Area 1, 5% for Area 2, and 10% for Area 3. The corresponding model-based output SNR estimates are 21.94, 17.92, and 13.55 dB, while the output SCR estimates are 18.42, 14.18, and 9.65 dB. From Area 1 to Area 3, SNR and SCR decrease by 8.39 and 8.77 dB, respectively. This comparison indicates weaker estimated target-to-background separation for the area with the highest moisture content. Because the three moisture values correspond to different test areas, other site properties may also contribute to the differences; the table alone does not isolate the causal effect of moisture. Moreover, its SNR and SCR values are model-based estimates, not a measured denoising evaluation on B-scans from all three areas. Cross-area robustness should therefore be assessed using measured B-scans from each area, with per-area target-preservation and clutter-suppression results and an area-held-out test.

The quantitative values reported for Figure 8 are image-level diagnostics of the displayed PNG triplet. They demonstrate that the proposed dataset yields a substantially smoother residual image than the FDTD-noise training set. Since a clean measured reference is not available for this field B-scan, these values are reported as no-reference residual-energy and texture indicators rather than PSNR or SSIM.

Compared with GAN-based methods, the main advantage of the proposed framework is controllability. GPR-GAN and multiscale-discrimination GANs can improve augmentation efficiency and visual realism, but adversarial training does not inherently identify whether a pixel belongs to direct coupling, target response, stochastic background, or multipath. In contrast, the proposed method constructs these components before transformer modulation and exports them after generation. Therefore, the generated dataset can support supervised denoising losses, target-preservation losses, clutter-specific suppression losses, and ablation studies using the same samples.

Compared with diffusion-based generation, the main advantage is generation efficiency and label availability. GPR-VMD-Diffusion improves the spectral and statistical consistency of simulated and real signals by using VMD mode fusion and diffusion recovery, which is valuable when high-fidelity distribution alignment is the primary objective. However, iterative diffusion sampling and VMD preprocessing increase the generation pipeline complexity. The proposed model does not attempt to replace high-fidelity electromagnetic simulation or diffusion recovery. Instead, it provides a faster route for producing physically interpretable denoising pairs and clutter labels at scale.

Compared with forward-model-guided methods such as FM-GAN, the proposed generator has a different target use case. FM-GAN learns the mapping from underground dielectric-constant maps to B-scans and is therefore useful for forward-model approximation. The method proposed in this paper is oriented toward denoising data construction: it does not require a complete dielectric map for every sample, and it directly controls target response, direct antenna coupling, stochastic clutter, and delayed multipath. This makes it more convenient when the objective is to train a denoising network that should suppress clutter while preserving weak target echoes.

In summary, the proposed framework gains its key advantage by explicitly decoupling and controlling physically interpretable GPR signal components. Unlike GAN-based methods, it directly constructs direct coupling, target response, stochastic clutter, and multipath, thus providing inherent controllability and native support for supervised denoising losses and ablation studies. Compared with diffusion-based generation, it trades high-fidelity distribution alignment for substantially higher efficiency and immediate label availability, eliminating iterative sampling and complex preprocessing. In contrast to forward-model-guided approaches such as FM-GAN, it is tailored for denoising data construction, directly manipulating individual signal components without requiring complete dielectric-constant maps. The current results provide a physically interpretable basis for scaling paired GPR denoising-data construction, but broad field robustness and site-independent generalization remain to be established through the larger multi-condition and multi-site experiments described above.

## 6. Conclusions

This paper presented a fast denoising dataset-generation method for GPR B-scans by combining a transformer network with an underground clutter physical model. The method was designed to address the lack of large, physically interpretable, and denoising-oriented GPR datasets. Instead of generating only image-level B-scans, each sample is exported as a structured data group containing the noisy B-scan, target mask, clean target response, direct-coupling clutter, stochastic background, and multipath clutter. This decomposition makes the generated data directly usable for supervised denoising, target-preserving loss design, clutter-specific suppression analysis, and ablation experiments.

The experimental results verified both the efficiency and the output capability of the proposed framework. A batch generation run produced 100 full-resolution 512×512 B-scans in 38.36 s on a CUDA-enabled GPU, equivalent to 2.61 B-scans/s and 15.64 exported maps/s. The generated dataset contained 600 primary maps and 157.3 million stored pixel values. The target-mask coverage remained within 10.08–13.84%, and all target and clutter components had non-zero energy across the dataset. The mean correlation between the generated B-scans and the reference image was −0.0210, with a maximum of 0.0779, showing that the generator used the reference only as a statistical style prior rather than as a pixel-level template.

Compared with representative GAN-based, diffusion-based, and forward-model-guided GPR generation methods, the proposed method provides a more suitable output structure for denoising dataset construction. It increases the directly exported products from one image-level B-scan to six maps per sample, provides five additional supervision or component maps, and explicitly separates three clutter components without adversarial or iterative diffusion sampling during generation. In the downstream comparison on the 1200-sample measured GPR dataset, the transformer network trained with the proposed dataset produced a smoother residual background and stronger clutter suppression than the network trained with FDTD data plus stochastic-noise augmentation. The controlled generator diagnostics remain based on a 100-sample synthetic run; the measured dataset size is 1200 samples, while independent multi-site and site-held-out generalization remain to be established.

The matched ablation further clarifies the scope of the transformer contribution. In its present training-free form, the component-specific transformer pathway produces small, bounded changes and modest average improvements in RFID and RIS, but its effect varies with initialization and is not uniformly favorable across all diagnostics. The transformer is therefore retained as the core architectural innovation for nonlocal, component-wise modulation rather than presented as a trained feature learner. Future work will train or deterministically calibrate this pathway so that its contribution can be made initialization-independent while preserving the physical decomposition and label structure of the generator.

The physical-validation results support the conclusion that the proposed framework offers an explicit efficiency–fidelity trade-off. It generated 100 full-resolution B-scans and their component labels in 38.36 s while maintaining a 4.70% propagation-delay MAPE and a 14.41% curvature MAPE over five FDTD conditions. This gain in dataset-construction efficiency is accompanied by a 44.50 dB target/direct amplitude error and a 45.53% multipath-delay MAPE. The method is therefore suitable for rapid, target-geometry-preserving and component-labeled denoising-data construction, but it is not a calibrated substitute for full-wave simulation. Future work will calibrate scattering amplitudes and replace the fixed multipath replicas with layer-dependent paths using raw multi-condition field measurements.

Future work will also investigate a grouped and energy-normalized parameterization of Equation (24), reducing the six independently tuned component gains to two or three constrained group-level coefficients and evaluating its robustness across survey sites and frequency bands.

The main limitation of the present validation is its restricted scale and field diversity: the quantitative study contains 100 generated B-scans and does not include a site-held-out denoising test across the three selected test areas. In future work, we will generate at least 1000 stratified samples, document the coverage of medium, moisture-related effective parameters, frequency, target depth, target geometry, and clutter strength, and evaluate the denoiser on independent measured B-scans from each of the three areas. A site-held-out protocol, per-site uncertainty estimates, and cross-frequency and cross-medium tests will be used to determine whether the component-labeled synthetic data transfer beyond the nominal configuration.

Our experiment demonstrates that the proposed generator can efficiently produce component-labeled GPR denoising data with controlled target, clutter, and multipath structure. The reported throughput, export integrity, reference-similarity diagnostics, component-level output counts, and qualitative five-band noise comparison are all reproducible from the supplied generator. The results also show that the proposed dataset yields a more effective training prior for transformer-based denoising than FDTD data with stochastic-noise augmentation because it produces stronger clutter suppression and a smoother residual background.

## Figures and Tables

**Table 1:** Nominal acquisition and target parameters declared by the proposed generator.

Parameter	Current Value	Interpretation
Image size	512×512 pixels	Exported B-scan resolution.
Nominal sampling rate	$10\times 10^{9}$ Hz	Acquisition metadata used by the generator configuration.
Scan-distance step	0.01 m	Lateral scan increment in the geometry record.
Antenna spacing	0.30 m	Transmitter–receiver separation.
Air gap	0.20 m	Nominal antenna-to-surface distance.
Wall/soil thickness	0.30 m	Nominal near-surface layer thickness.
Wall/soil relative permittivity	5.0	Effective dielectric parameter declared by the geometry model.
Cavity/target-region thickness	0.55 m	Nominal deeper-region parameter in the geometry record.
Target count	1–3 per B-scan	Number of sampled hyperbolic responses.
Normalized target depth	0.24–0.67	Depth range in normalized image coordinates, not calibrated metres.

**Table 2:** Main data products exported by the proposed generator for each B-scan sample.

Data Product	Array Size in the 100-Sample Run	Role in Denoising Dataset Construction
images	100×1×512×512	Noisy B-scan samples containing target echoes and all clutter components.
targets	100×1×512×512	Binary target masks used for target-preserving denoising and target-aware loss design.
clean	100×1×512×512	Clean target-response maps used as supervised denoising references.
direct	100×1×512×512	Direct antenna-coupling component, corresponding to early coherent leakage.
random	100×1×512×512	Random background, row-correlated, band-limited, and impulsive clutter component.
multipath	100×1×512×512	Interface-induced delayed and virtual-target multipath component.

**Table 3:** Diagnostics of the 100 generated B-scan samples.

Metric	Mean	Std.	Min.	Max.
MSE to reference	0.01267	0.00023	0.01216	0.01325
Correlation to reference	−0.0210	0.0362	−0.1207	0.0779
Target-mask coverage	0.1174	0.0085	0.1008	0.1384
Target energy	0.4297	0.2286	0.1458	0.9335
Direct-coupling energy	0.4744	0.1035	0.1909	0.6833
Random-clutter energy	0.5100	0.0220	0.4558	0.5655
Multipath-clutter energy	0.2461	0.0457	0.1574	0.3671

**Table 4:** Matched ablation of the physics-only, fusion-only, and complete transformer–fusion configurations. For the complete model, the final row reports the mean ± standard deviation across five neural initialization seeds. Lower RFID, pairwise MS-SSIM, R-LPIPS, and MSE are preferred; paired MS-SSIM measures similarity to the matched physics-only output and is reported as a diagnostic rather than a quality objective. ↓ indicate that the smaller indicator is preferred. ↑ indicate that the larger indicator is preferred.

Configuration	Seed	RFID ↓	RIS ↑	MS-SSIM ↓	R-LPIPS ↓	MSE to Physics ↓	MS-SSIM to Physics
Physics only	–	3560.204	1.577	0.7013	1.152	0	1.0000
Fusion only	101	3453.650	1.694	0.6941	1.148	0.000019	0.9952
Full model	101	3464.468	1.745	0.7038	1.151	0.000026	0.9936
Full model	202	3560.967	1.438	0.7213	1.154	0.000027	0.9942
Full model	303	3614.093	1.632	0.7150	1.162	0.000034	0.9930
Full model	404	3562.247	1.744	0.7142	1.155	0.000013	0.9971
Full model	505	3456.920	1.671	0.6930	1.150	0.000021	0.9940
Full model	Mean ± std.	3531.739±68.356	1.646±0.126	0.7094±0.0111	1.154±0.005	0.000024±0.000008	0.9944±0.0016

**Table 5:** Physical-feature comparison between scene-conditioned analytical generation and raw gprMax/FDTD simulations. Delay is referenced to the coherent direct arrival; amplitude columns report envelope ratios in decibels. Each entry is generated/FDTD.

Scene	$\epsilon_{r}$ Path	Target	$t_{0}$ (ns)	*a* (ns $m^{-2}$)	$A_{t}/A_{d}$ (dB)	$\Delta t_{\mathrm{mp}}$ (ns)	$A_{\mathrm{mp}}/A_{p}$ (dB)
M1T7S1	9	free-space sphere	14.566/14.832	8.919/10.117	−20.461/−73.209	2.744/9.665	−5.193/−3.257
M2T5S1	16	PVC sphere	19.426/19.739	11.893/13.515	−23.982/−76.034	2.744/8.028	−5.193/−3.137
M4T2S1	9/16	PEC cylinder	16.418/16.776	11.443/13.281	−13.970/−53.831	2.744/3.177	−5.193/−0.307
M5T10S1	6	PEC box	12.677/11.167	7.022/9.197	−14.886/−51.181	2.753/1.877	−5.193/−0.675
M6T6S1	27	PEC sphere	25.232/26.411	15.449/13.954	−14.440/−55.996	2.753/3.928	−5.193/3.426
Mean error			0.725 ns	1.665 ns $m^{-2}$	44.50 dB	2.938 ns	4.40 dB
MAPE where defined			4.70%	14.41%	–	45.53%	–

**Table 6:** Radar-domain image-quality comparison after reimplementation under the identical local protocol. ↓ indicate that the smaller indicator is preferred. ↑ indicate that the larger indicator is preferred.

Method	Basis	RFID ↓	RIS ↑	Pairwise MS-SSIM ↓	R-LPIPS ↓
GPR-GAN [9]	100	2287.987	1.098	0.876	1.396
GAN with multiscale discrimination [10]	100	1989.631	1.196	0.784	1.387
GPR-VMD-Diffusion [15]	100	1878.692	1.245	0.716	1.318
FM-GAN [13]	100	2293.085	1.107	0.771	1.392
FDTD + stochastic acquisition noise	32	2047.702	1.050	0.220	1.317
Proposed model	100	1703.823	1.286	0.715	1.293

**Table 7:** Model-based estimates of output SNR and SCR for the moisture contents of three selected test areas.

Test Area	Volumetric Water Content (%)	Output SNR (dB)	Output SCR (dB)
1	1	21.94	18.42
2	5	17.92	14.18
3	10	13.55	9.65

## Data Availability

The raw data supporting the conclusions of this article will be made available by the authors on request.

## References

[1] Skartados E., Kostavelis I., Giakoumis D., Tzovaras D. (2019). Automatic Subsurface Map Generation Based on GPR Data Processing. Proceedings of the 2019 IEEE SmartWorld, Ubiquitous Intelligence & Computing, Advanced & Trusted Computing, Scalable Computing & Communications, Cloud & Big Data Computing, Internet of People and Smart City Innovation (SmartWorld/SCALCOM/UIC/ATC/CBDCom/IOP/SCI).

[2] Ning K., Zhou C., Yu Z. (2023). Segment anything model for subgrade distress detection with GPR. Proceedings of the 2023 IEEE 3rd International Conference on Data Science and Computer Application (ICDSCA).

[3] Hou F., Qiao B., Dong J., Ma Z. (2024). S-CycleGAN: A Novel Target Signature Segmentation Method for GPR Image Interpretation. IEEE Geosci. Remote Sens. Lett..

[4] Kang M., Kaji S., Lee S.Y., Kim T., Ryu H.H., Choi S. (2026). Shape-Aware Topological Representation for Pipeline Hyperbola Detection in GPR Data. IEEE Sens. J..

[5] Zhang G., Zhou Z., Wan C., Ding Z., Wu Z., Xie L., Xue S. (2025). Substructural damage identification in a digital twin framework using heterogeneous response reconstruction. Adv. Struct. Eng..

[6] Zhang G., Hou J., Wan C., Li J., Xie L., Xue S. (2025). Non-contact vision-based response reconstruction and reinforcement learning guided evolutionary algorithm for substructural condition assessment. Mech. Syst. Signal Process..

[7] Schorlemer J., Jebramcik J., Baer C., Rolfes I., Schulz C. (2022). A Statistical FDFD Simulator for the Generation of Labeled Training Data Sets in the Context of Humanitarian Demining using GPR. Proceedings of the 2022 IEEE MTT-S International Conference on Numerical Electromagnetic and Multiphysics Modeling and Optimization (NEMO).

[8] Zhao D., Guo G., Ni Z.K., Pan J., Yan K., Fang G. (2023). WAEGAN: A GANs-Based Data Augmentation Method for GPR Data. IEEE Geosci. Remote Sens. Lett..

[9] Xiong H., Li J., Li Z., Zhang Z. (2024). GPR-GAN: A Ground-Penetrating Radar Data Generative Adversarial Network. IEEE Trans. Geosci. Remote Sens..

[10] Wang B., Li K., Wu S., Chen P. (2024). GPR B-Scan Image Augmentation via GAN With Multiscale Discrimination Strategy. IEEE Trans. Geosci. Remote Sens..

[11] Tian H., Bai X., Zhu X., Arun P.V., Mi J., Zhao D. (2025). CIGGAN: A Ground-Penetrating Radar Image Generation Method Based on Feature Fusion. IEEE Trans. Geosci. Remote Sens..

[12] Hao K., Yang B., Guo C., Wang J. (2025). An Improved DC-GAN-Based Approach for Ground Penetrating Radar Image Generation. Proceedings of the 2025 International Applied Computational Electromagnetics Society Symposium (ACES-China).

[13] Xu T., Yuan D., Yang G., Li B., Fan D. (2024). FM-GAN: Forward Modeling Network for Generating Approaching-Reality B-Scans of GPR Pipelines With Transformer Tuning. IEEE Trans. Geosci. Remote Sens..

[14] Chen G., Hu C., Hou L., Ge S., Zhang Y., Wang C., Fu L.Y., Zhao Y. (2025). Elevating GPR Imaging With DDPM: A Comprehensive Study on Synthetic Data Generation. IEEE Trans. Geosci. Remote Sens..

[15] Li F., Yang F., Shi W., Peng S., Li Q. (2025). GPR-VMD-Diffusion: A Data Generation Algorithm Using Modes Features of GPR Signals for Road Underground Targets. Proceedings of the 2025 IEEE Radar Conference (RadarConf25).

[16] Pang Z., Jiang Y., Zhong J., Lei W. (2026). Forward-Guided and Reverse-Resampling Diffusion Model for GPR B-Scan Data Restoration and Generation. IEEE Trans. Geosci. Remote Sens..

